# Post-radiation cranial fasciitis in a pediatric medulloblastoma survivor: A case report and systematic review

**DOI:** 10.1016/j.ijscr.2025.111695

**Published:** 2025-07-16

**Authors:** Mohsen Koosha, Roya Mostafavi, Azin Kheradmand, Jina Behjati

**Affiliations:** a Department of Neurosurgery, Imam Hossein Hospital, Shahid Beheshti University of Medical Sciences, Tehran, Iran; b Department of Pathology, School of Medicine, Shahid Beheshti University of Medical Sciences, Tehran, Iran

**Keywords:** Case report, Child, Cranioplasty, Fasciitis, Radiotherapy, Scalp

## Abstract

**Introduction and importance:**

Cranial fasciitis (CF) is a rare, benign fibroproliferative lesion primarily affecting children. Post-radiation CF is particularly uncommon, and has been reported in only seven previous cases. Its presentation often mimics malignancy, with nonspecific preoperative findings complicating the diagnosis, therefore necessitating early intervention.

**Case presentation:**

We report a 12-year-old male with a history of medulloblastoma treated with chemoradiotherapy four years ago, who presented with a progressive right temporo-occipital scalp mass. Imaging showed an extradural mass with calvarial bone erosion. Complete surgical excision was performed, and histopathology confirmed CF. No recurrence was observed at six-month follow-up.

**Clinical discussion:**

Post-radiation CF is an extremely rare complication of radiotherapy. Due to its rapid growth, bony invasion, and occasional intracranial extension, it can be misdiagnosed as a radiation-induced neoplasm. Given the overlap in clinical and radiologic features with malignancies such as meningioma or sarcoma, histopathological confirmation is essential. Unlike neoplasms, CF follows a benign course, and complete surgical excision is often curative.

**Conclusion:**

Post-radiation CF should be considered in children with prior radiotherapy presenting with scalp masses. Early diagnosis and surgical intervention are crucial for avoiding unnecessary treatments and ensuring favorable outcomes.

## Highlights

• Cranial fasciitis can develop as a rare complication of radiotherapy in pediatric brain tumor survivors
• Post-radiation cranial fasciitis mimics malignancy with potential transcranial extension and bone erosion
• Complete surgical excision in most cases is curative with excellent prognosis despite aggressive appearance
• Early diagnosis prevents unnecessary treatments in this benign fibroproliferative lesion

## Graphical abstract

**Figure:**
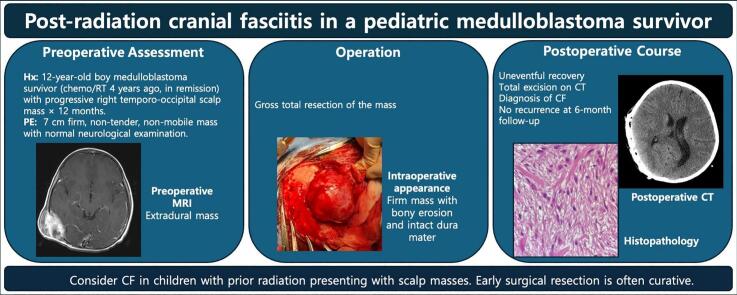

## 1 Introduction

Cranial fasciitis (CF) is a rare, non-cancerous fibroproliferative lesion that primarily affects the scalp and skull of children, commonly under the age of six [1,2]. CF is a form of nodular fasciitis, a growth of fibroblastic cells arising from superficial and deep fascia [3]. While nodular fasciitis and CF are histologically similar, CF originates from galea aponeurosis, pericranium, dura, or the skull and primarily affects children [[3], [4], [5]]. CF usually presents as a single, rapidly growing, firm, non-tender mass on the scalp, more common in the temporal and parietal regions [6]. Although most cases are limited to soft tissue, some have transcranial extensions with bone erosion and dural involvement, which can lead to mass effect on underlying brain structures and need prompt evaluation and surgical intervention [2]. The exact etiology of this lesion remains unclear; however, based on reported cases, it has been associated with trauma, prior radiation exposure, hereditary factors, or idiopathic causes [2].

To date, approximately 80 cases of pediatric CF have been reported [2], with only seven cases secondary to radiation [5,[7], [8], [9], [10], [11], [12]]. The clinical challenge lies in distinguishing benign cranial fasciitis from malignant tumors, as both can present with alarming rapid growth and lytic bone destruction on imaging [2]. This diagnostic uncertainty necessitates surgical intervention for a definitive pathological diagnosis, which is crucial for confirming the excellent prognosis of CF and avoiding inappropriate cancer therapies.

Here, we present the case of a 12-year-old boy with a history of medulloblastoma treated with chemotherapy and radiation, who developed a progressively enlarging scalp mass in the temporo-occipital region. We discuss our diagnostic approach, surgical management, and postoperative outcome, followed by a systematic review of the literature on post-radiation CF to better characterize this rare entity. This case report has been reported in line with the SCARE 2025 checklist [13].

## 2 Case presentation

### 2.1 History and physical examination

A 12-year-old boy, presented to our neurosurgery clinic for evaluation of a progressively enlarging scalp lesion, which had been growing over the past 12 months.

The patient himself was entirely asymptomatic. He specifically denied any headaches, dizziness, seizures, changes in vision or hearing, or any focal neurological deficits such as weakness, numbness, or tingling. The mass was not painful or tender to the touch. His past medical history was significant for medulloblastoma, diagnosed four years prior, which had been treated with a full course of chemotherapy and radiotherapy. He completed his last radiation treatment three years ago and had been in complete remission since. There was no family history of neurofibromatosis or similar cutaneous or cranial lesions.

On physical examination, the patient was alert and cooperative. Inspection of the scalp revealed a well-circumscribed, firm, non-mobile mass in the right temporo-occipital region, measuring 7 cm in its largest diameter. The overlying skin was intact, with no discoloration, ulceration, or inflammatory signs. A comprehensive neurological assessment was performed and was entirely unremarkable.

### 2.2 Assessment

**Fig. 1:**
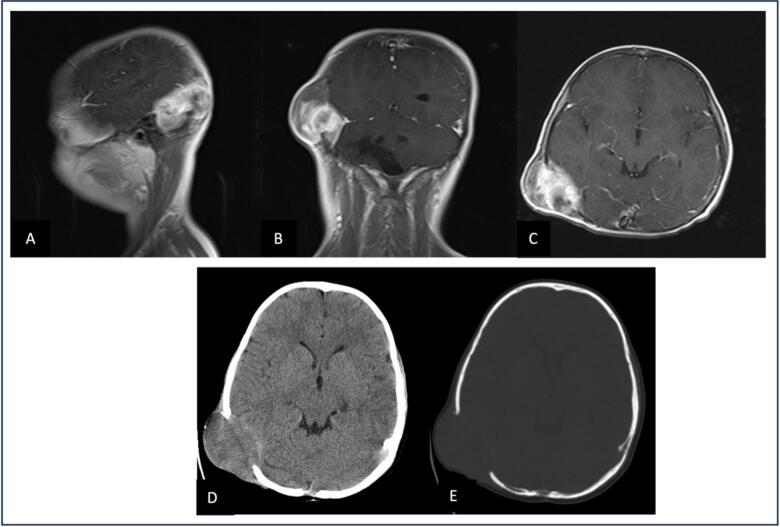
Preoperative imaging (A) T1-weighted MRI sagittal view with contrast enhancement showing a right temporo-occipital mass with heterogeneous signal intensity extending to the skull. (B) T1-weighted MRI coronal view with contrast enhancement demonstrating the extra-axial nature of the lesion with enhancement and adjacent bone involvement. (C) T1-weighted MRI axial view with contrast enhancement clearly delineating the right temporo-occipital mass. (D) CT brain window axial view revealing the soft tissue component of the mass with isodense appearance. (E) CT bone window axial view highlighting the lytic bone lesion with destruction of the right temporo-occipital bone.

For further evaluation, computed tomography (CT) and magnetic resonance imaging (MRI) were performed, which demonstrated a well-defined posterior fossa lesion measuring 5.0 × 4.0 × 2.0 cm (Fig. 1). The lesion appeared extradural and extra-axial. CT scan showed significant lytic lesions of the cranium adjacent to the mass. Additionally, all laboratory tests were within normal limits, and systemic workup showed no evidence of other organ involvement, supporting the lesion as a primary rather than metastatic process.

### 2.3 Operation

**Fig. 2:**
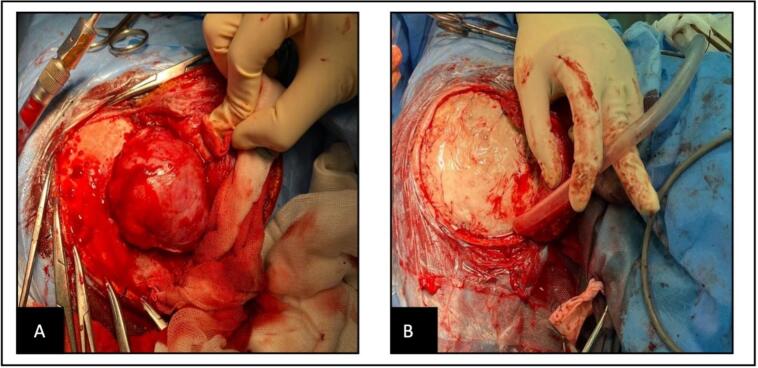
(A) Intraoperative appearance of the mass following craniotomy of the occipital bone, (B) The lesion has been dissected from the dura, with no signs of dural invasion.

Under general anesthesia, a microsurgical resection was performed by our senior author via a right-sided suboccipital retrosigmoid craniotomy with Mayfield head fixation in the lateral position. Intraoperatively, extensive erosion of the inner cranial table was noted, with a firm pink mass invading the right transverse and sigmoid dural venous sinuses (Fig. 2). Following complete resection of the mass, cranioplasty was performed using a 10 × 10 cm titanium mesh.

### 2.4 Outcome

**Fig. 3:**
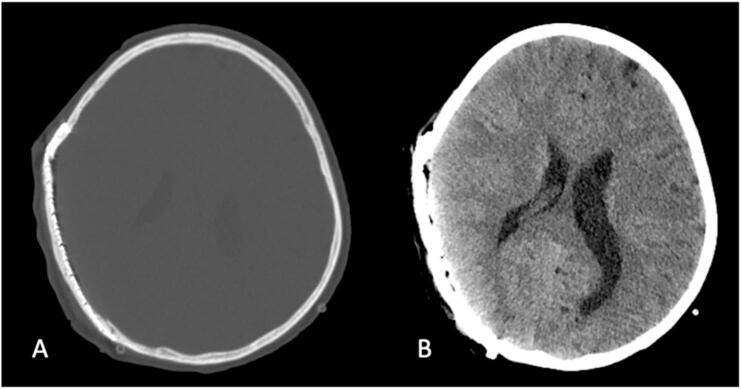
Postoperative computed tomography showing complete mass removal with titanium mesh cranioplasty, (A) bone window and (B) brain window.

The patient had an uneventful postoperative recovery and was discharged home four days after surgery without any complications. Postoperative imaging confirmed total excision of the mass (Fig. 3).

**Fig. 4:**
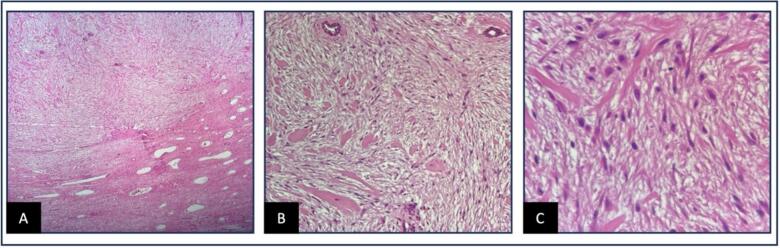
The examination reveals a proliferation of spindle-shaped fibroblasts and myofibroblasts embedded within a collagenous and myxoid stroma (hematoxylin and eosin, x40), (hematoxylin and eosin, x100), and (hematoxylin and eosin, x400).

Histopathological examination of the biopsy revealed hypocellular, bland spindle cells arranged in a storiform and whirling pattern, interspersed with thick collagen bundles (Fig. 4). Immunohistochemical (IHC) analysis revealed that the spindle cells were positive for vimentin, smooth muscle actin (SMA), and desmin, negative for S100 protein, and showed nuclear positivity for beta-catenin. The proliferation rate, assessed by Ki-67, was <1 %. These findings, together with the clinical and radiologic context, were consistent with a diagnosis of CF.

At his 6-month postoperative follow-up, the patient remained neurologically asymptomatic, and the surgical wound was healed. Our long-term monitoring protocol includes regular clinical examinations and serial imaging for surveillance. The patient is scheduled for his next follow-up MRI at one year post-surgery, with the frequency of subsequent scans to be determined by clinical and radiological findings.

## 3 Systematic review

### 3.1 Method

A systematic review was conducted following the Preferred Reporting Items for Systematic Reviews and Meta-Analyses (PRISMA) guidelines [14] to identify all reported cases of CF following radiotherapy or radiation exposure.

### 3.2 Eligibility criteria

All articles written in English that reported cases of CF with a history of radiotherapy were included in this systematic review. Publications such as comments, editorials, opinion pieces, review articles without case reports, book chapters, and conference abstracts were excluded. Additionally, studies were excluded if they lacked patient-specific data, did not confirm a history of radiation exposure, or reported fasciitis in anatomical locations other than the cranium.

### 3.3 Search strategy

A comprehensive literature search was conducted in PubMed, Scopus, Embase, and Web of Science to identify relevant studies. The search strategy included free-text keywords including: “cranial fasciitis”, “cranial fasciitis AND skull”, “nodular fasciitis AND cranial”, and “cranial fasciitis AND scalp”, to ensure broad retrieval of cases. MeSH terms were not used as “cranial fasciitis” is not indexed as a MeSH term. Exact search strings for each database are provided in supplementary material. The search was performed on February 22, 2025. After retrieving the initial results, duplicates were removed, and two independent reviewers screened titles and abstracts to identify relevant articles. Disagreements were resolved through discussion until consensus was reached. This was followed by a full-text review based on the predefined eligibility criteria. Finally, studies that met the inclusion criteria proceeded to data extraction for further analysis.

### 3.4 Risk of bias assessment

Risk of bias assessment was not performed as all included studies were single case reports, which inherently carry high risk of bias.

## 4 Results

### 4.1 Study selection

**Fig. 5:**
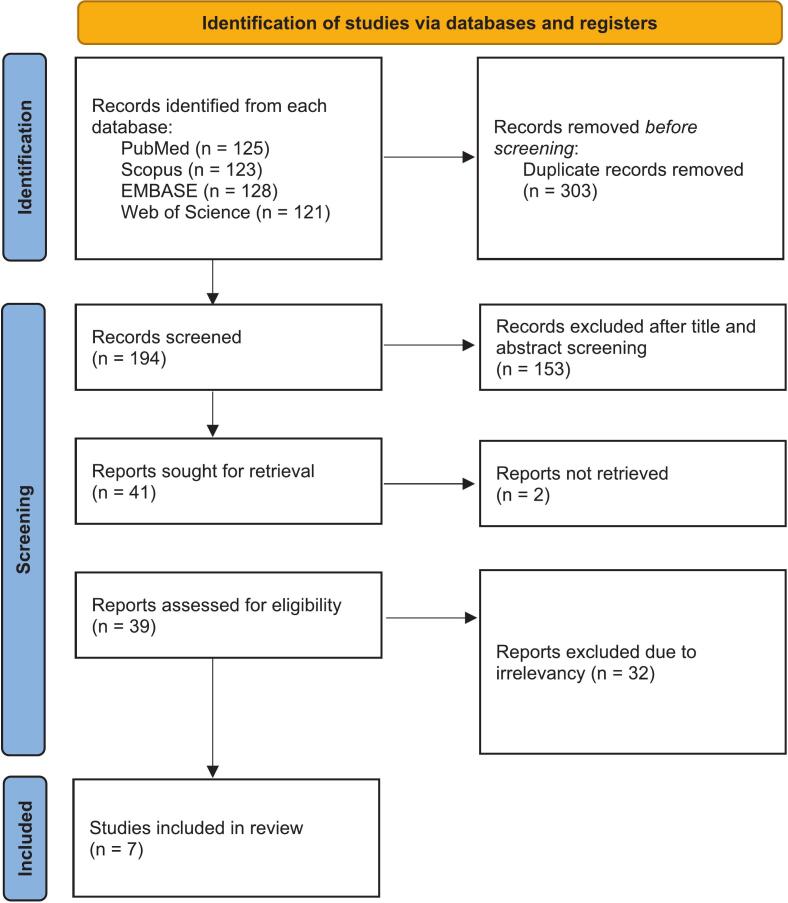
PRISMA flow diagram.

The initial search across PubMed, Scopus, Embase, and Web of Science yielded 497 results. After duplicate removal, 194 unique articles remained. These articles underwent title and abstract screening, followed by a full-text review based on the predefined eligibility criteria. After applying the inclusion and exclusion criteria, seven studies reporting CF with a history of radiotherapy were included in the final review. The PRISMA flow diagram illustrating the study selection process is presented in Fig. 5.

### 4.2 Qualitative synthesis

A summary of previously reported cases of post-radiation CF is presented in Table 1. Among the eight cases, five involved female patients. All cases were children, with age at diagnosis ranging from 2 to 16 years (median 12.5 years). The primary disease in six cases, including ours, was medulloblastoma, and primitive neuroectodermal tumor (PNET) and rhabdomyosarcoma were reported in the remaining two cases [7,8]. The interval between radiation therapy and CF presentation ranged from 1 to 3.5 years, with a median of 1.4 years. The tumor location varied across studies. None of the patients had a history of trauma. All cases had bony invasion with or without dural involvement, and in all but one case [12], gross total resection (GTR) was achieved. No case of recurrence was reported in the available follow-up data.

**Table 1:** Summary of reported cases of cranial fasciitis following radiation therapy.

Author, Year	Age (yrs), Sex	Primary disease	Interval between RT and CF detection	Hx of trauma	Tumor location	Origin	Bony invasion	Treatment	F/U duration and recurrence
Sarangarajan [10], 1999	14, F	Medulloblastoma	1	No	Temporoparietal	Dura	Yes, with dural invasion	GTR	>5 yrs., No recurrence
Longatti [11], 2004	11, M	Medulloblastoma	3.5	No	Midline parietal	External layer of dura	Yes	GTR	12 m, No recurrence
Summers [9], 2007	16, F	Medulloblastoma	1.17	No	Lt occipital	Scalp soft tissue	Yes, with dural adhesion	GTR	12 m, No recurrence
Johnson [7], 2008	11, F	PNET	3	No	Rt parietal	Epidural space	Yes, without dural invasion	GTR	Not reported
Wu [5], 2013	13, F	Medulloblastoma	1	No	Rt occipital diploic space	diploe	Yes	GTR	12 m, no recurrence
Hattab [8], 2014	2.7, F	Rhabdomyosarcoma	1.7	No	Lt parietal	Dura	Yes, with dural adhesion	GTR	8 yrs., No recurrence
Malik [12], 2022	2, M	Medulloblastoma	1	No	Occipital	Dura	Yes, with dura invasion	STR	10 m, residual tumor stable
Present study	12, M	Medulloblastoma	2	No	Temporo-occipital	Dura	Yes/without dural invasion	GTR	No recurrence

**Abbreviations:** CF: cranial fasciitis, F: female, F/U: follow-up, GTR: gross total resection, Hx: history, Lt: left, M: male, PNET: primitive neuroectodermal tumor, Rt: right, yrs.: years, m: month.

## 5 Discussion

We report a case of a 12-year-old boy with a history of radiotherapy for medulloblastoma presenting with a progressively enlarging scalp mass in the right temporo-occipital region. Our differential diagnosis, considering the rapid progression and radiation exposure, included meningioma, metastasis of the primary tumor, or a reactive process. Since the preoperative diagnosis was uncertain, the patient underwent complete surgical excision, and a biopsy was taken to rule out neoplasm. Histopathological findings were consistent with CF.

CF is a rare, benign fibroproliferative lesion, first described by Enzinger in 1980 as a subset of nodular fasciitis [15]. CF primarily affects children between 3 weeks and 6 years old [2]. The reported male-to-female ratio suggests a slight male predominance, although findings across studies vary [2,12]. CF can occur anywhere on the skull, but temporal and parietal regions are the most common sites [16]. These lesions can arise from scalp soft tissue, deep fascia, and periosteum, and may even extend intracranially with dural invasions [16]. These lesions are usually firm, painless, and rapidly growing, with sizes ranging from a few centimeters to large ones exceeding 5 cm, often raising concerns for soft tissue neoplasms [2]. The clinical symptoms vary based on the location and the extent of invasion [16]. These lesions are typically solitary, though cases with multiple lesions have been documented [17].

The exact cause of CF remains unclear, but it is generally categorized as trauma-related, post-radiation, hereditary, or idiopathic, with trauma being the most frequently reported trigger [2]. Since our patient had a history of prior radiotherapy, we suspect radiation as the underlying cause of CF. Aside from our patient, seven other cases of post-radiation CF have been reported in the literature [5,[7], [8], [9], [10], [11], [12]]. However, the link between radiation and CF remains controversial, as the exact mechanism by which radiation may trigger CF is not well understood. Most of these studies point out the temporal association with CF occurring after radiation exposure [5,[7], [8], [9], [10], [11], [12]]. Additionally, a spatial correlation has been noted, especially when CF occurs within the irradiated field but away from the surgical site, suggesting the role of radiation rather than surgical trauma in the development of CF. [5,7,10] Longatti et al. performed a cytogenetic analysis of CF lesions, revealing multiple chromosomal aberrations, including clonal and nonclonal changes [11]. This pattern was consistent with radiation-induced lesions observed in experimental models, supporting radiation as a potential cause [11]. Notably, most cases, including ours, had a shorter interval between radiotherapy and CF detection, differentiating it from radiation-induced neoplasms, which have longer latency periods [5].

Preoperative imaging for CF includes CT and MRI for optimal evaluation and surgical planning. CT scans help detect lytic bone lesions, sclerosis, and ossification, particularly when the tumor invades the skull, making them useful for planning craniotomy and cranioplasty [6]. MRI provides superior soft tissue contrast, showing tumor structure and invasion extent [2]. T1-weighted imaging typically reveals hypointensity with vivid enhancement and a central non-enhancing region (as seen in our case) while T2-weighted imaging shows hyperintensity in the central non-enhancing area corresponding to myxoid matrix and hypointense regions reflecting fibrous tissue [6]. However, these imaging findings are nonspecific and can be seen in various intracranial pathologies; therefore, histological examination is required for definitive diagnosis of CF.

Histologically, CF is characterized by spindle- or stellate-shaped fibroblasts and myofibroblasts proliferating within a hyaline and myxoid stroma [5]. In our case, histopathological examination revealed the same morphological appearance, with low Ki-67 expression (<1 %), rare mitoses, and myofibroblastic differentiation (vimentin/SMA/desmin positive, S100 negative), ruling out high-grade sarcoma and neurogenic tumors. The differential diagnosis for pediatric cranial spindle cell lesions includes cranial fasciitis, desmoid fibromatosis, and dermatofibrosarcoma protuberans [6,18]. Desmoid fibromatosis was excluded despite nuclear beta-catenin positivity due to the absence of characteristic fascicular architecture and myxoid stroma, extreme rarity of this condition in pediatric cranial locations, and rapid growth inconsistent with desmoid behavior [19]. Dermatofibrosarcoma protuberans was ruled out by the deep location and storiform pattern without honeycomb architecture [6]. The nuclear beta-catenin positivity, while unusual for cranial fasciitis, has been documented in other reported cases in the literature [20]. Combined with the characteristic morphological features, clinical presentation, and exclusion of other entities, this supports the diagnosis of cranial fasciitis and warrants clinical follow-up and potential molecular testing.

The primary treatment for CF is complete surgical excision, which is typically curative, and chemotherapy or radiation is not recommended [2]. Although spontaneous regression has been observed, surgery is preferred, especially in cases with intracranial involvement [5]. However, observation or alternative treatments may be more appropriate for lesions in surgically challenging locations (e.g., the petrous bone) [2]. The surgical approach depends on tumor location and the extent of invasion. In cases with extensive bony involvement, as seen in our case, skull reconstruction is necessary. We removed the involved bone and reconstructed the skull with titanium mesh cranioplasty.

With complete excision, recurrence risk is low, though a systematic review in 2019 identified six recurrent cases, which developed between 6 months and 4 years postoperatively [2]. In such cases, re-excision is recommended, and malignancy should be ruled out [2]. In our case, at 6-month follow-up, MRI showed no recurrence of either medulloblastoma or CF, and the patient had an uncomplicated recovery with no new symptoms. From the patient's perspective, the postoperative recovery was uneventful, and both he and his parents reported relief and satisfaction with the surgical outcome.

This study has several limitations. First, the follow-up duration was limited to six months, which may be insufficient to detect late recurrences or long-term complications, especially given the indolent nature of some fibroproliferative lesions. Second, although histopathological and immunohistochemical analyses were performed, molecular profiling, such as testing for USP6 or COL1A1-CAMTA1 gene fusions, was not conducted. This limits insight into the potential genetic or neoplastic nature of the lesion and prevents comparison with emerging molecular classifications of CF. Third, the rarity of post-radiation CF restricts the generalizability of our findings, as conclusions are drawn from a small number of reported cases with limited heterogeneity. Lastly, the systematic review was restricted to English-language literature and relied on published case reports, which may introduce publication bias and underreporting relevant cases. As this report is based on a single patient, its conclusions are inherently limited. To build a more comprehensive understanding of this rare condition, larger studies are crucial.

## 6 Conclusion

We present a rare case of CF occurring after radiotherapy for medulloblastoma. This diagnosis should be considered in children with a history of prior radiation, particularly when they present with a rapidly growing scalp mass. Definitive diagnosis relies on histological examination to distinguish CF from malignant tumors. Due to its rapid growth and potential for intracranial extension, early surgical resection is recommended. Fortunately, complete excision is typically curative, with a low recurrence rate, emphasizing the importance of timely recognition and intervention.

## Abbreviations

CF: Cranial Fasciitis
CT: Computed Tomography
MRI: Magnetic Resonance Imaging
IHC: Immunohistochemistry
SMA: Smooth Muscle Actin
GTR: Gross Total Resection
PNET: Primitive Neuroectodermal Tumor

## Author contribution

Mohsen Koosha: Conceptualization, supervision, validation, review and editing of the manuscript.

Jina Behjati: Formal analysis, Investigation, methodology, writing of the original draft, validation, review and editing of the manuscript.

Roya Mostafavi: Data curation, investigation, validation, review and editing of the manuscript.

Azin Kheradmand: Investigation, validation, review and editing of the manuscript.

All authors have read and agreed to the published version of the manuscript.

## Patient consent

Written informed consent was obtained from the patient's legal guardians to publish this case report and accompanying images. A copy of the written consent form is available for review by the editor-in-chief of this journal upon request.

## Ethical approval

Our institution exempts/waives ethical approval for de-identified case reports.

## Guarantor

Dr. Mohsen Koosha accepts all responsibility for this article.

## Declaration of Generative AI and AI-assisted technologies in the writing process

During the preparation of this work the authors used Grammarly in order to correct grammar and improve writing. After using this tool/service, the authors reviewed and edited the content as needed and take full responsibility for the content of the publication. All inputs to the AI tool consisted solely of general academic writing content that did not contain patient information, personal identifiers, or data subject to GDPR/HIPAA regulations.

## Funding

No funding was received for this article.

## Declaration of competing interest

All authors declare that they have no conflicts of interest.

## Appendix A Supplementary data

Supplementary material Image 1
