# Supplementary material for: Post-radiation cranial fasciitis in a pediatric medulloblastoma survivor: A case report and systematic review
Source: Int J Surg Case Rep. 2025 Jul 16;133:111695. doi: 10.1016/j.ijscr.2025.111695 (PMC12284691; doi:10.1016/j.ijscr.2025.111695)
Supplement: Supplementary file 1 — Supplementary material [file mmc1.docx]

Search Strategy

| Database | Search String | Number of results |
| --- | --- | --- |
| PubMed  2/22/2025 | ("cranial fasciitis" OR ("nodular fasciitis" AND cranial) OR ("cranial fasciitis" AND scalp)) | 125 |
| Scopus  2/22/2025 | TITLE-ABS-KEY("cranial fasciitis" OR "cranial fasciitis AND skull" OR "nodular fasciitis AND cranial" OR "cranial fasciitis AND scalp") | 123 |
| EMBASE  2/22/2025 | ('cranial fasciitis' OR 'cranial fasciitis AND skull' OR 'nodular fasciitis AND cranial' OR 'cranial fasciitis AND scalp') | 128 |
| Web of Science  2/22/2025 | TS=("cranial fasciitis" OR "cranial fasciitis AND skull" OR "nodular fasciitis AND cranial" OR "cranial fasciitis AND scalp") | 121 |
| Google Scholar  2/22/2025 | "cranial fasciitis" OR "cranial fasciitis AND skull" OR "nodular fasciitis AND cranial" OR "cranial fasciitis AND scalp" | 678 |
